# The Biologically-Based Bias of Personality Disorder Diagnosis

**DOI:** 10.3389/fpsyg.2016.01293

**Published:** 2016-08-26

**Authors:** Steven Hertler

**Affiliations:** Department of Psychology, College of New RochelleNew Rochelle, NY, USA

**Keywords:** life history, personality, diagnosis, disorder, DSM, evolution

## Introduction

More than previous versions, the American Psychiatric Association's (2013) fifth edition of the *Diagnostic and Statistical Manual of Mental Disorders* (DSM-V) formally specified the nature of a personality disorder. Going beyond specific personality disorders and their respective criteria sets, are the *General Criteria for Personality Disorder* (GCPD) provided in section III on page 761 of DSM-V. Much of this list simply specifies that impairments must be enduring-transcending time, place, medical status, and developmental period. It is only the first two, criterion *A* and criterion *B*, which positively define a personality disorder. Specifically, criterion *A* states that there must be “moderate or greater impairment in personality functioning,” and criterion *B* states that there must be “one or more pathological personality traits.” Thereafter, on page 762, the DSM attempts to define and operationalize the concepts of *pathological personality traits* and *impaired personality functioning.* First, DSM-V operationalizes pathological personality traits in so far as five are listed: (1) negative affectivity, (2) detachment, (3) antagonism, (4) disinhibition, and (5) psychoticism. Second, in describing *personality functioning*, there is mention of disturbances in *self* and *interpersonal* functioning, which are taken to “constitute the core of personality psychopathology.” DSM-V proceeds to parse self and interpersonal functioning further: “Self-functioning involves identity and self-direction; interpersonal functioning involves empathy and intimacy.”

All such efforts are commendable improvements on what would otherwise have been imprecise concepts liable to hold different meanings for different readers. Precision is gained by exposition. Yet, precision invites criticism. With implicit assumptions giving way to explicit assertions, as collected across various tables, terms, and operational definitions within the GCPD, personality disorder diagnosis can be recognized as relativistic. When clinical judgment, diagnostic tradition and psychiatric authority are exchanged for empirical research, one finds personality variables like those embodied in the GCPD unassociated with deficits in vocation, mating success, finance, and health (Ullrich et al., 2007; Gutiérrez et al., 2013; Vall et al., 2015). At any point along the continuum of personality, instead of unalloyed deficits and beneficial traits, humans experience fitness relevant trade-offs (Nettle, 2006; Brumbach et al., 2009), as do damselflies (Rodin and Johansson, 2004), rainbow trout (Schjolden et al., 2006), house mice (Rauw, 2006) and a host of other animals displaying rudimentary personalities (Bell, 2007; Wolf et al., 2007; Biro and Stamps, 2008; Dammhahn, 2012; Favreau et al., 2014). What is true for traits may be true for personalities. As previously reviewed (Hertler, 2015a), psychopathy and obsessive compulsive personality disorder have both been described as strategic, evolved patterns, rather than dysfunctional personality disorders. Moreover, a recent study found personality extremes consistent with DSM-V disorders to be sexually selected via female choice (Vall et al., 2015). With the fact of relativism being elsewhere treated (Hertler, 2015b), it suffices to state that the GCPD rests on clinical assumptions of pathology which do not appear to equate to impairments in evolutionarily relevant life outcomes. Herein, it is not the question of relativism itself that is pursued, but the nature of that relativism. GCPD criteria are neither arbitrarily relativistic nor culturally relativistic; instead they show a particular bias, comprehensible only from a life history evolutionary vantage point.

### Bringing a life history evolutionary perspective to bear

Life history evolution is a coherent sub-discipline of evolutionary biology that originated with the study of variation across seven developmental trait clusters, among which were lifespan, maturation rate, and brood size. Life histories are distributed across a gradient from *fast* or *r-selected*, to *slow* or *K-selected.* Most basically, and confined to the classic biomarkers upon which life history theory was grounded, *fast* and *slow* relate to the pace of development. Fast or *r*-selected species mature quickly, breed much, and die young, whereas slow or *K*-selected species show the opposite pattern, living long enough to support extensive prenatal growth and thereafter lavish resources on a small number of long-lived young. Contrast the elephant with the rabbit. In number, size, and care of young, as in longevity, growth and maturation rate, these animals are extraordinarily dissimilar. Life history variation, though greatest between species, is present within species; including the human species. Within the past three decades, life history evolution has been particularly successful in logically grouping altruism and affiliation, risk aversion and inhibition, as well as future oriented thought and delay of gratification (Figueredo et al., 2006; Jonason and Tost, 2010; McDonald et al., 2012; Hertler, 2015a), showing them to be *K*-selected. Opposite these, across a continuum, are *r*-selected antagonism, sensation seeking, disinhibition, and an orientation to the present. The original biological, as well as the aforementioned psychological and social life history variables, are collectively calibrated by mortality; specifically the rate, predictability and controllability of mortality. In *K*-selected regimes, mortality is rare, predictable and controllable; in *r*-selected regimes, mortality is common, unpredictable, and uncontrollable (Schechter and Francis, 2010). As investing in a few, slow growing, late maturing young is impractical and maladaptive under an *r*-selected regime with high, and highly unpredictable mortality, so is excessive altruism, risk-aversion or future orientation. There is not sufficient time or safety to capitalize on investment. So, life history creates a sort of time relevant biology organized around mortality.

### The general criteria for personality disorder as an inventory of fast life history markers

When the GCPD is viewed from a life history perspective, it becomes apparent that the *r*-selected tail of the life history distribution is being wontedly pathologized. The lion's share of GCPD components are indeed fast life history traits. GCPD is first broadly conceived of as *self-impairmen*t, which relates to limited self-direction and goal-pursuit, both of which mark the present oriented opportunism of the *r*-strategist (Figueredo and Rushton, 2009). Under *self-impairment* also come the personality traits of psychoticism and disinhibition. Within psychoticism, as conceived by Eysenck, are the traits of impulsivity, sensation-seeking and risk-taking, all of which are *r*-selected. The *self-impairment* component of the GCPD marks as pathological, a present focused time orientation and the behaviors that follow from such an orientation. The *r*-selected are persons on the tail end of a distribution adapted best to novel, changing and unpredictable conditions. Impulsivity, sensation seeking, disinhibition, risk-taking and presentism, rather than being indicative of pathological *self-impairment* as per the GCPD, are behavioral dispositions that follow logically, and function suitably, in the context of unsafe and unpredictable environments (Griskevicius et al., 2011).

The *General Criteria for Personality Disorder* (GCPD) is secondly broadly conceived of as *interpersonal-impairment*, which relates to deficits in empathetic response, durable intimate connections, and mutual interpersonal regard, all of which mark the instrumental selfishness of the *r*-strategist (Olderbak and Figueredo, 2010). Under *interpersonal-impairment* also come the personality traits of negative affectivity, detachment and antagonism. Within a life history framework, however, negative affectivity, detachment and antagonism are simply manifestations of a fast life history which expresses “less mutualistic and more antagonistic” behavior in response to “unstable, unpredictable, and uncontrollable” conditions (Sherman et al., 2013). In contrast to *r*-strategists, *K*-strategists, “prefer long-term and cooperative social as well as sexual relationships, which are easier and more profitable to maintain in their characteristically more stable, predictable, and controllable environments” (Figueredo and Rushton, 2009). Which is expressed relates not at all to questions of superiority or inferiority, or more pertinently, ordered or disordered personality. Instrumental or exploitative exchanges, restricted empathy and altruism, and short-term mating, with its emphasis of quantity above quality of offspring, rather than being indicative of pathological *interpersonal-impairment* as per the GCPD, are interpersonal strategies that compete effectively with their opposite, most especially in unsafe and unpredictable environments (Brumbach et al., 2009).

## The GCPD's correspondence with life history measures and factors

Life history measures and factors run parallel to the GCPD. The *Life Experiences Questionnaire*, the *Self-Control Schedule, The Barrett Impulsivity Scale*, the *Mating Effort Scale* and the *Mate Value Inventory* all informed the construction of explicit life history measures like the *Arizona Life History Battery* (Figueredo et al., 2004, 2006; Gladden et al., 2009). Not only do life history batteries overlap significantly with the GCPD, so do aggregated life history variables like the *K-Factor, Covitality, General Factor of Personality* (GFP), and the *Super-K Factor*. The *K-Factor*, for instance, predicts attachment, mating effort, Machiavellianism, and risk-propensity (Figueredo et al., 2005). Further still, nearly uniformly related to the GCPD, depressions in the following factors were found to be inter-correlated, heritable markers of *r*-selected life histories: (1) quality relationships with mother, father, spouse, children; (2) family support; (3) altruism toward kin; (4) friends support; (5) altruism toward non-kin; (6) close relationship quality; (7) communitarian beliefs; (8) religiosity; (9) financial status; (10) advice seeking; (11) foresight/anticipation; (12) insight into past; (13) primary control/persistence; (14) flexible/positive reappraisal; (15) self-directedness/planning (Figueredo et al., 2004). As will be obvious, the first eight life history correlates equate closely to the GCPD's *interpersonal-impairment* component, with its emphasis on affiliation and reciprocation. In turn, most of the remaining seven life history correlates relate to the GCPD's *self-impairment* component, with its emphasis on restraint and future oriented planning.

## Conclusions

Life histories, whether *r* or *K* selected, are heritable and developmentally sensitive patterns across which there is intra-population variation. This variation, though moderated environmentally, is evolutionarily created and maintained (Réale et al., 2010). Again, neither end of the life history distribution is absolutely better or worse; rather each is better or worse relative to a specified mortality regime (Chisholm, 1999). In other words, as mortality waxes and wanes in randomness and rate, so will the fitness of GCPD related life history traits, such as altruism, foresight, persistence and planning. All these traits assume an extended future where what is sown can be reaped, whether it is a hard-earned reputation for cooperation, or a long-term goal achieved (Geronimus, 1987). The *K* strategist is evolutionarily and developmentally prepared to maximize long-term benefit and altruistic cooperation in predictable and controllable environments. The *r* strategist is evolutionarily and developmentally prepared to maximize resource extraction and immediate interpersonal gains in unpredictable and uncontrollable environments (Olderbak et al., 2014). Lamentably, the GCPD conflates *strategic difference* and *pathological dysfunction* because it was created in the absence of a life history perspective, or apparently in the absence of any evolutionary grounding.

Assuredly, there is no conscious effort to use psychiatric diagnosis of personality disorders as an agent of social control or a tool of discrimination. Nevertheless, adrift from evolutionary moorings, GCPD standards have exposed a non-arbitrary strain of relativism; they too closely resemble a compilation of perceived *r*-failings compiled by *K*-strategists. Indeed, it is not only that the *r*-strategist is likely to function as the identified patient, but that the *K*-strategist is likely to be the diagnosing clinician. Pursuing this notion, there is some evidence that clinicians more consistently live by, and evince, *contemporary middle-class North American Values* (Greene, 1985; Garb, 1997; Hall, 2001; Samuels, 2004), which are *K*-selected correlates (Figueredo et al., 2006). Similarly, relating to the longevity and bodily maintenance that marks the *K*-selected (Kaplan et al., 2000; Flatt et al., 2013), physicians as a group elevate on health behaviors (Frank, 2004) and longevity (Frank et al., 2000), while having significantly lower all-cause mortality rates compared to the general population (Torre et al., 2005; Aasland et al., 2011). Additionally, the brain and behavioral *K*-selected correlates of intelligence (Rushton, 2004) and education (Figueredo et al., 2006) are directly relevant to patently educated and disproportionately intelligent psychiatrists and psychologists culled from the general population via admissions tests (SAT, GRE, MCAT) graduate programs, internships, residencies, as well as post-doctoral and licensure requirements. In turn, education is associated with (Stentz et al., 2016) delayed (Pew Research Center, 2016) and limited reproduction (Weeden et al., 2006), both foundational *K*-selected biomarkers (Chisholm, 1999).

Being that diagnosing clinicians may well be disproportionately *K*-selected, it then becomes important to understand that *K* strategists don't just evince the *K* strategy, they actively shape the environment toward the stability and predictability wherein it best operates. The *K*-selected are “overrepresented among the rule-enforcers” (Gladden et al., 2009), often serving to promulgate and reinforce social norms (Figueredo et al., 2006, 2007; Sherman et al., 2013). This is a process of niche construction wherein the environment is accommodated to the needs of the organism, not taken as it is found. *K*-selected rule creation and enforcement, like the bird building a nest, or the beaver building a dam (Hughs, 2012), is indicative of an extended phenotype (Dawkins, 1999). One end of the life history gradient working against the other is then an example of competition in which the weapons are not claws and teeth, but agents of social control like prisons and sanctions. Legislating against and locking up those that express *r* selected extremes may be defensible within the criminal justice system. Competition of this nature, however, is not patently appropriate for the diagnostic endeavor to the extent that it has pretensions to objectively identifying instances of pathology. As race, class and sex now are, it follows that diagnostic categories and decisions should then be scrutinized for life history related bias. With respect specifically to the GCPD, it might be removed in favor of a life history informed system that eschews diagnosing r-selected pathology in favor of identifying its incongruous expression in *K*-selected contexts.

## Author contributions

The author confirms being the sole contributor of this work and approved it for publication.

### Conflict of interest statement

The author declares that the research was conducted in the absence of any commercial or financial relationships that could be construed as a potential conflict of interest.
